# Radiomics in Breast Imaging: Future Development

**DOI:** 10.3390/jpm13050862

**Published:** 2023-05-20

**Authors:** Alessandra Panico, Gianluca Gatta, Antonio Salvia, Graziella Di Grezia, Noemi Fico, Vincenzo Cuccurullo

**Affiliations:** 1 Radiology Division, Department of Precision Medicine, Università della Campania “Luigi Vanvitelli”, 80138 Naples, Italy; alessandrapanico1@gmail.com (A.P.); gianluca.gatta@unicampania.it (G.G.); antoniosalvia89@gmail.com (A.S.); 2 Radiology Division, ASL Avellino, 83100 Avellino, Italy; graziella.digrezia@gmail.com; 3 Department of Physics “Ettore Pancini”, Università di Napoli Federico II, 80126 Naples, Italy; noemifico@gmail.com; 4 Nuclear Medicine Unit, Department of Precision Medicine, Università della Campania “Luigi Vanvitelli”, 80138 Naples, Italy

**Keywords:** radiomics, breast imaging, artificial intelligence

## Abstract

Breast cancer is the most common and most commonly diagnosed non-skin cancer in women. There are several risk factors related to habits and heredity, and screening is essential to reduce the incidence of mortality. Thanks to screening and increased awareness among women, most breast cancers are diagnosed at an early stage, increasing the chances of cure and survival. Regular screening is essential. Mammography is currently the gold standard for breast cancer diagnosis. In mammography, we can encounter problems with the sensitivity of the instrument; in fact, in the case of a high density of glands, the ability to detect small masses is reduced. In fact, in some cases, the lesion may not be particularly evident, it may be hidden, and it is possible to incur false negatives as partial details that may escape the radiologist’s eye. The problem is, therefore, substantial, and it makes sense to look for techniques that can increase the quality of diagnosis. In recent years, innovative techniques based on artificial intelligence have been used in this regard, which are able to see where the human eye cannot reach. In this paper, we can see the application of radiomics in mammography.

## 1. Introduction

Breast cancer is the most common and the most diagnosed non-skin cancer in women: breast cancer is detected approximately as one case in every three malignancies cases (29%). Breast cancer is the leading cause of death at any age, accounting for the 29% of death before the age of 50. The 29% of deaths by breast cancer splits as follows: 21% of the deaths occur between the ages of 50 and 69, and 14% of the deaths occur after the age of 70 [1,2].

**Figure 1:**
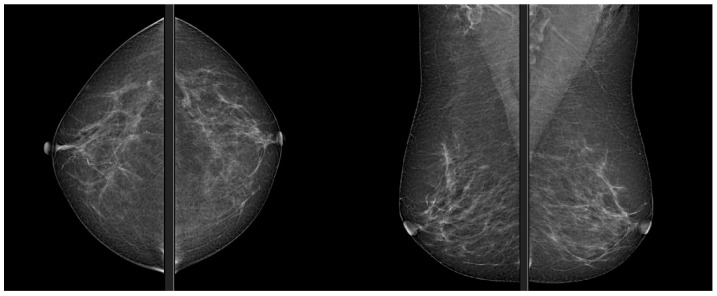
Mammography, CC and MLO views.

Mammography is the reference technique in breast cancer radiological diagnosis, and it is recognized as the main screening tool for its effectiveness, Figure 1. Its diagnostic efficacy is clearly superior to clinical examination, allowing the identification of small anomalies, especially in recognizing microcalcifications [1]. According to the directives of the Ministry of Health, breast cancer screening is aimed at women between the ages of 50 and 69 and consists of performing a mammogram every 2 years.

Early diagnosis allows the implementation of conservative therapeutic surgery and a significant reduction in mortality to ensure a better prognosis for women. Unfortunately, some breast tumors are unrecognizable in mammography or in women with high glandular density breasts as well as for those who are undergoing surgery. The presence of small masses can be masked; thus, the sensitivity of mammography is significantly reduced, as well as its diagnostic reliability.

Mammography is the reference technique in radio-breast diagnosis. Its diagnostic efficacy is clearly superior to clinical examination, allowing the identification of small anomalies, especially in recognizing microcalcifications. According to the directives of the Ministry of Health, breast cancer screening is aimed at women between the ages of 50 and 69 and consists of performing a mammogram every 2 years.

Early diagnosis allows the implementation of conservative therapeutic surgery and a significant reduction in mortality. Unfortunately, some breast tumors are unrecognizable on mammography or in women with high glandular density breasts, as the presence of small masses can be masked, thus the sensitivity of mammography is significantly reduced [3].

This is why the fight against breast cancer seeks new allies capable of minimizing errors: the field of radiomics is an interesting one. It is important to discuss the basics to understand this new growing field.

Radiomics, in fact, has as its objectives both to make the reading of the image as objective as possible and to make a contribution to the visual analysis of the radiologist. There are, in fact, objective limitations in image readings that can be overcome with the intervention of analysis by artificial intelligence. The features identified in the ROIs of mammographic images can be of various classes. We have a first subdivision: first order and higher order. In the first-order features, there are the mean, median, standard deviation of Hounsfield values, entropy, i.e., the degree of unpredictability of the distribution of gray levels, skewness indicating histogram symmetry, and Kurtosis. Second-order features, on the other hand, include descriptors from the contrast group, such as dissimilarity and homogeneity; order-related descriptors, such as angular momentum, energy, and entropy; statistical descriptors that analyze the frequencies of pairs of values, such as mean, standard deviation, and correlation; and descriptors that analyze the differences in gray levels between each image element and those immediately bordering it, such as coarseness, contrast, and activity [4,5,6]. In this paper, we will talk about artificial intelligence, in the most general terms, hinting at what are the techniques of Machine Learning and Deep Learning, with a mention of neural networks. We will discuss in detail how radiomics can be effectively adapted for use in mammography.

The paper wants to illustrate how Machine Learning techniques have been developed in radiomics to propose a personalized diagnosis and treatment to breast cancer patients. It is a review about how radiomics works on radiological images and what is the purpose of it.

## 2. Artificial Intelligence

The term Artificial Intelligence (AI) was introduced in 1956 by mathematician John McCarthy, during a research conference in Dartmouth, where AI was decreed as an academic research area. However, it could be argued that the creation of the field began earlier with Alan Turing, who developed the homonymous test, and it is even possible that its origin predates this. Over time, AI has taken on different definitions, and one of the most common describes it as the “branch of computer science that deals with the simulation of intelligent behavior in computers”.

More specifically, it is possible to define AI as the branch of informatic science dedicated to the creation of algorithms capable of solving problems without being explicitly programmed for all their specificities [7].

However, a universal definition is still the subject of debate among experts today. The term AI encompasses many specific areas and approaches, such as computer-assisted diagnosis (CAD), Machine Learning, radiomics, noise reduction, and image acquisition optimization. Artificial intelligence can be divided into strong and weak. Narrow (also called weak) artificial intelligence, in which a computer can perform a very specific task as well as or even better than humans (for example, IBM’s Watson computer that beat two champions by Jeopardy in 2011), uses programs that can solve or analyze single problems or perform specific jobs through a gradual learning process. General (strong) artificial intelligence is defined by when a computer goes beyond specific tasks to perform higher-order processes by emulating human thought processes [8,9].

Strong AI instead uses systems that obtain a sort of self-awareness through the emulation of human thought and are able to reason and solve problems, guaranteeing superior learning and decision-making capabilities. In this sense, AI can be understood as a set of tools and programs that make software "smarter" that an external observer thinks that the output is generated by a human being. In fact, it works in a similar way to normal human brain functions during regular tasks, such as common sense reasoning, opinion forming, or social behavior [1,8,9].

The history of AI began in the last century. There were many innovations in the study of collective phenomena, which have led scholars to create new mathematical models useful for the advancement of technology. In particular, interest in biological neural networks has laid the foundations for the study and understanding of collective phenomena such as artificial neural networks, which were inspired by biological models. These studies laid the foundations for cybernetics and AI. The first wave started with cybernetics in the 1940s–1960s, with the development of the theories of biological learning with McCulloch and Pitts in 1943, in which the single neuron was used as a binary threshold unit, a basic logical unit of the brain, capable of performing simple functions. The model considered a single neuron that received from other units, different signals, and they were summed in a weighted way, providing a single result. The synapses evaluated the signal by multiplying it by the associated weight $w_{ij}$.

Synapses contain information on the long/short term memory of a network, as the corresponding $w_{ij}$ weights are determined by the learning process. The output obtained is comparable to the activation potential transmitted along the axon of a neuron (spike), which will have unique behavior: if the output exceeds the activation threshold from the neuron, a spike will start and reach the connected units

The signal evolves according to the equation:

(1) $$n_{i}\left(t+1\right)=\theta [\sum w_{ij}n_{j}\left(t\right)-\mu_{i}]$$

this equation defines the dynamics according to which the system evolves. From a time t to a time t + 1, time t, in fact, has discrete values, with one unit of elapsed time per processing step.

- $n_{i}$ can take the values 1 or 0 and represents the state of an active or switched-off neuron, respectively;
- θ(x) is the activation function, in this case the step function of Heavside;
- $w_{ij}$ is a weight function called the synaptic coefficient, related to the interaction and strength of the synapse between the ith neuron and the jth neuron. It can be positive if it represents an excitatory synapse, otherwise negative if inhibitory; these weights could be set by the human operator;
- $\mu_{i}$ is the parameter indicating the threshold value for unit i. The weighted sum of the inputs must reach or exceed the threshold for activation of the neuron.

The McCulloch and Pitts model is a simplistic model of the biological neuron, omitting certain neurobiological and mathematical complications. The peculiarity of the model, however, lies in the fact that the neuron is modeled as a basic unit of the brain, analogous to a computer processing unit; it processes information but with substantial differences, which allow the brain network to be more efficient on average due to the neural interconnection that allows parallel and distributed information processing, a mode of execution opposite to Von Neuman’s conventional type of processing. A fundamental evolution in this first model, which contributed to the birth of AI, occurred with the implementation of the neurobiological property of plasticity, which was theorized by the neuroscientist Santiago Ramón y Cajal and later postulated by Hebb.

Plasticity allows the network to learn and store through synaptic structural changes, and this is the basis of the learning and information retrieval process. This process is called Hebbian learning, the main idea of which is based on the following hypothesis: “if a neuron A is close enough to a neuron B to contribute repeatedly and durably to its excitation, then a process of growth or metabolic change takes place in both neurons such that the e cacy of A in exciting B is increased”.

Hebb’s postulate was based on Hopfield’s model of 1982, a model that described the processes of memorization and learning, taking into account the neurophysiology of the individual neuron and the dynamics of the network that behaves as a dynamic system of a ferromagnetic type (Ising’s model 1919), where the dynamics of the system evolves towards a basin of attraction, a stable point in the network, i.e., a memorized pattern.

Hopfield started from McCulloch and Pitts’ model (1), which described the activity of the single neuron. The modifications made to (1) concerned a convection on the activated and deactivated unit $S_{i}=\pm 1$ and the dynamics are rewritten via the function sgn(x), obtaining

(2) $$S_{i}:=sgn\left(\sum_{j}w_{ij}S_{j}-\theta_{i}\right)$$

Under the assumption of random patterns $\theta_{i}=0$. The dynamics of the network will then be described by the following dynamics equation, whereby each unit is allowed to choose to update itself. Finally, for the pattern to be effectively stored, i.e., to be an attractor of the dynamics, the necessary condition is that it is a fixed point of the dynamics, i.e., that the evolution of the network evolves towards the attractor of the dynamics $\xi_{i}^{\mu }$.

Initializing the network in the state $S_{i}=\xi_{i}^{\mu }$ connections $w_{ij}$ are chosen in order for the network to remain in the state $S_{i}=\xi_{i}^{\mu }$. This is the stability condition of the μ -th memory system.

Synaptic coefficients $w_{ij}$ represent the interaction between neurons ij; thus, the function can take on positive or negative values depending on whether the synapse is excitatory or inhibitory, and these weight functions vary so that attractors of dynamics are generated to allow the process of retrieval of stored information. In the Hopfield model, the weights $w_{ij}$ are the result of the learning rule inspired by Hebb’s studies, where $\xi_{i}^{\mu }$ is the component of the µth pattern to be stored.

In 1952, Hodgkin and Huxley, starting with experimental observations on the axon of the giant squid, developed a mathematical model of the electrophysiological processes associated with the cell membrane. Cragg and Temerley, in 1955, reformulated the McCulloch–Pitts model, likening the state of the neuron, active or inactive, to the positions of the orientable spins in a magnetic system, and, in 1961, Caianiello introduced statistical methods for treating the model, incorporating the Hebbian learning hypothesis.

In 1958, the psychologist Rosenblatt first introduced the concept of the perceptron, whose units were organized in two layers with feed-forward connections: this was an early neural network model useful for the classification and recognition of shapes, with variable weight functions, which enabled learning. The perceptron became the first model capable of learning the weights defining different categories, given the input examples of each category. A similar model was proposed with Widrow and Hoff in the early 1960s, who created the Adaline (ADAptive LInear NEuron) network, again based on the McCulloch–Pitts model, which differed from the perceptron in the weight functions used by the network. The Adaline algorithm was a convergent classification model based on the use of linear adaptive artificial neurons. The training algorithm used to adapt the ADALINE weights was a special case of an algorithm called stochastic gradient descent.

Today, neuroscience is considered an important source of inspiration for Deep Learning researchers, and we are currently able to derive some broad guidelines from neuroscience. The basic idea of having many computational units that only become intelligent through their interactions with each other is inspired by the brain.

In summary, artificial intelligence algorithms, and, in particular, Deep Learning, aim to assist humans in solving a problem or solving a problem without the help of human input. The exponential increase in computational processing and memory capacity has opened up great potential for AI to handle much larger data sets, including those required in radiology and, more specifically, in breast imaging [5,10,11].

### 2.1. Machine Learning

Machine Learning is a term that was introduced by Arthur Samuel in 1959 to define a field of AI, in which computers learn automatically from the accumulation of data. It is a specific practical application of informatic science and mathematics that allows computers to extrapolate information from observed patterns without being explicitly programmed.

In general, computers are able to solve mathematical problems in a very short time that a human being would face with difficulty. However, at the same time, tasks that we accomplish every day naturally and with little difficulty are a real challenge for a computer. Some examples are the visual recognition of objects or words. Programming proves to be ineffective in addressing this type of problem. Therefore, algorithms are used to simulate the way human beings learn. Advances in Machine Learning might have an important impact on our understanding of human learning and teaching strategies, and it could give a contribution to understanding how biological learning systems could provide important guidance to Machine Learning, and vice versa [12,13,14].

Machine Learning research aims to develop computer systems that automatically improve their performance through experience. The aim of Machine Learning research is to produce computer systems that learn to operate in various fields, solving problems of high complexity.

Machine Learning studies algorithms that learn from experience: it is a generic system that is capable of automatically acquiring experience, given the initial information about the patterns and behaviors of the system’s components, assisting and apprenticing human beings performing such tasks. The definition of a learning problem is: “A computer program is said to learn from experience E with respect to a class of tasks T and a performance measure P, if its performance at tasks in T, as measured by P, improves with experience E”. (Tom Mitchell).

In general, Machine Learning is useful for natural language processing, robotics, genetic sequence analysis, software engineering, image processing, and the acquisition of problem-solving strategies. This extends the capacity for collaborative human–machine problem solving. The main capability of any learning program is the ability to generalize, i.e., to transfer knowledge learned in one situation and extend it to other situations [15,16,17].

Machine Learning is a subfield of artificial intelligence with the goal of developing algorithms capable of learning from data automatically. In particular, an artificially intelligent agent needs to be able to recognize objects in its surroundings and predict the behavior of its environment in order to make informed choices. Therefore, techniques in Machine Learning tend to be more focused on prediction rather than estimation [18,19,20]. Estimation and prediction are related; they often lead to different approaches. If we consider some observable quantity x of the system, we are studying relating to some parameters θ, we could consider p(x|θ) as the probability of observing *x* give θ. We perform an experiment to obtain a dataset X and use these data to fit the model, where fitting mean finds the best explanation for the data. For example, if we consider the method of least squares, the estimated parameters maximize the probability of observing the data

(3) $$\hat{\theta }=argmax_{\theta }\left\{p\left(X|\theta \right)\right\}$$

Estimation problems are concerned with the accuracy of $\hat{\theta }$. Prediction problems are concerned with the ability to predict new observations the accuracy of p(x|θ).

The methods that allow the computer to learn without being explicitly programmed are, therefore, called Machine Learning, or Machine Learning (AA). Machine Learning algorithms evolve as data exposure increases; they are not based merely on rules, but improve with experience: they learn to give specific answers by evaluating large amounts of data [21,22,23].

One use of artificial neural networks relates to the ability to store and recognize complex patterns, thanks to an adaptive memory capacity. Applications are based on learning processes. Learning is a process whereby the free parameters of a neural network are adapted, through a process of stimulation, to the environment in which it is embedded.

Machine Learning algorithms are typically classified into four broad categories, also called paradigms:

- **Unsupervised learning**: the agent learns patterns from the structure of the inputs provided even without any explicit feedback. The network learns autonomously; it is presented with inputs, which will be classified by the network by identifying similarities and classifying them. Unsupervised learning deals with finding patterns and structures in unlabeled data. These techniques include clustering, dimensionality reduction, and generative modeling.
- **Reinforcement learning**: the agent learns from a series of reinforcements, rewards, or punishments obtained from the previous activity. In reinforcement learning, an agent learns by interacting with an environment and changing its behavior to maximize its reward;
- **Semi-supervised learning**: less output is provided to the agent. It is critical to validating the performance of AI verifying the accuracy of Machine Learning outcomes.
- **Supervised learning**: the agent learns the output provided. The goal is to extract a general rule that correctly combines the input with the output; the network is trained by means of a teacher, there is a set of data, i.e., examples of both input and output, useful for training the network, and each input is assigned an ideal output configuration (target) so that the network can learn to deduce the relationship that binds them. The network will have to adjust its free parameters to best emulate the teacher. It is then trained by an algorithm, typically back-propagation, capable of minimizing the training-related error. The ultimate goal of the network is to predict the outcome, when there is no predefined output, based on a small number of matching examples; this is learning from labeled data. The most common supervised learning tasks include classification and regression.

### 2.2. Deep Learning

Machine Learning systems are used to identify objects in images, transcribe speech into text, match news items, etc. These applications use techniques called Deep Learning that can process unstructured data by autonomously identifying distinctive features of the analyzed systems, unlike Machine Learning techniques. Learning by representation is a set of methods that allows a machine to be fed with raw data and automatically discover the representations needed for detection or classification.

Deep Learning (DL) is a subset of Machine Learning, and it is the base of most artificial intelligence tools for the interpretation of images. It is defined as a set of methods of representation learning, which is based on different levels of representation, corresponding to hierarchies of factors or concepts characteristics, where the high-level concepts are defined on the basis of those of the bottom. This means that the computer has multiple levels of interconnected algorithms, and these are layered in hierarchies of importance, such as more or less significant data.

These layers accumulate data from inputs and provide output that can change step-by-step once the AI system learns new capabilities. Multilayer algorithms form large artificial neural networks, which are composed of thousands to millions nodes or units connected by links that propagate activation from one unit to another, and each link activation is weighted by a numerical value that determines the strength of the connection [8,23,24]. Deep Learning combines computing power and a structure similar to that of neural networks. Deep Learning methods are representation learning methods with multiple levels of representation, obtained by composing simple, non-linear modules that each transform the representation at one level, from the raw input, into a representation at a higher level; as the transformations are composed, increasingly complex functions can be learned. Deep Learning thus allows the computer to build complex concepts from simpler ones.

The quintessential example of a Deep Learning model is the multilayer perceptron (MLP), which is a mathematical function that maps a series of input values into output values. The main function is formed by the composition of many simpler functions. We can think of each application of a different mathematical function as providing a new representation of the input.

Another perspective is to consider that depth allows a computer to learn a computer program in several stages by executing a series of instructions in parallel. Networks with greater depth can execute multiple instructions in sequence.

The modern term ”deep learning” is related to the more principle of learning multiple levels of composition.

These methods are at the forefront in speech recognition, image recognition, visual object recognition, object detection, and many other fields such as drug discovery and genomics. Deep Learning is making great progress in solving problems that for many years resisted the best attempts of the artificial intelligence community.

### 2.3. Neural Networks

Some of the earliest learning algorithms that we know today were intended as computational models of biological learning, consequently, one of the names by which Deep Learning has been called is artificial neural networks (ANNs). Deep Learning is based on using computational systems called artificial neural networks. The architecture of artificial neural networks was inspired by biological models of the human brain, and was born with the aim of reproducing activities such as image recognition, language understanding, etc. Although the types of neural networks used for Machine Learning have sometimes been used to understand brain functions (Hinton and Shallice, 1991), they are generally not designed to be realistic models of biological functions.

Artificial neural networks must be trained using training datasets from which the network learns, combining learned functionalities to make decisions [8,9].

A neural network is made up of nodes, called neurons, which, based on the input they receive, change their state, active or inactive, and send a certain output. The outputs of some neurons are connected to the inputs of others, forming a network, through weighted and directed links. Convolutional Neural Networks are a type of neural network that are used for image analysis. They are called convolutional because at least one layer of the network uses the convolution operation on the inputs: the system, which receives images as input, uses convolutions as input levels [24,25]. Specifically, they are useful in the analysis of medical images, as they are able to recognize their configuration.

What makes RNCs so effective in image recognition is the ability they have to compose general characteristics, such as the presence of edges and light or dark spots, etc., into more complex shapes until they recognize the complete object. What makes this progressive composition of image characteristics possible is the multi-layered structure of the network [26]. An important application is in medical imaging, especially in breast radiology: in this context, Deep Learning is used for image segmentation, for the automatic labeling of images with the corresponding area or disease such as a mass, the diagnosis of diseases (computer-aided detection), in the field of radiomics (correlation of features of medical images with clinical outcomes), to make a quantitative measurement of breast volume density to capture the foreseeable risk of masking breast cancer, and to predict a woman’s risk of carrying a high-risk genetic mutation, etc. [26].

In this case, the convolutional layers of the dense neural network are integrated with each other and allow the extraction of intrinsic characteristics of the images; thus, allowing the obtainment of information on the type of breast lesion and its management as well as in the patient’s prognosis [27]. Neural networks usually consist, at least initially, of hand-labeled image data sets used by the algorithm to enhance its adaptation to match the fundamental truth.

Once a network has been trained using a training dataset, it would then be tested using a different dataset (validation dataset), designed to evaluate the model’s fit to the new data. In this phase, it is common to observe an “overfitting” of the model. Yamashita et al. described overfitting as the situation “in which a model learns the specific statistical regularities of the training set, i.e., it ends up memorizing the irrelevant noise instead of learning the signal and, therefore, performs less well on a new set of next data”. The consequence of overfitting is that the network will not be generalizable to data never seen before and will be the source of more errors of interpretation than those that occurred in the training dataset [24].

The best solution to reduce overfitting is to run multiple training and test cycles on different datasets, gradually improving network performance and allowing the accuracy and generalizability of the algorithm to be assessed before the algorithm is released for general use. Another solution is so-called "data augmentation", which means modifying the training data by adding some variability so that the model does not see exactly the same inputs from the training dataset during the training iterations [8,24,25].

Machine and Deep Learning algorithms provide powerful modeling tools to mine the huge amount of image data available, reveal underlying complex biological mechanisms, and make personalized precision cancer diagnosis and treatment planning possible [28,29,30,31]. Radiomics is an emerging area in quantitative image analysis that aims to relate large-scale extracted imaging information to clinical and biological endpoints. The human eye is limited, and some information could be lost, while the approach of radiomics utilizes methods differently. Quantitative imaging features, called also “radiomic features”, can provide richer information about intensity, shape, size, or volume and the texture of tumor phenotypes using different imaging modalities such as CT, PET, US, and MR imaging.

In combination with Machine Learning or Deep Learning, it makes an enormous contribution to personalized cancer treatments based on medical images. Specifically, the radiomic approach consists of combining radiomic feature extraction with Machine Learning, with the aim of detecting and diagnosing cancer or to automatically contour the lesion, typically using supervised methods trained on a set of features computed from multimodal images. The voxels were labeled as cancerous or non-cancerous using a Support Vector Machine classifier. Radiomics, in fact, cannot provide invasive information on the components of the tumor phenotype for multiple three-dimensional lesions at multiple time points during and beyond the course of treatment. It could happen by applying Machine Learning methods to radiomic features extracted from medical images. It is possible to macroscopically decode the phenotype of many physio-pathological structures and, in theory, solve the inverse problem of deducing the genotype from the phenotype, providing valuable diagnostic, prognostic, or predictive information.

However, quantitative imaging research is complex. In particular, the field of radiomics requires renewed attention to optimal study design/reporting practices and the standardization of image acquisition, feature calculation, and rigorous statistical analysis in order for the field to progress. It is used to detect benign masses or malignant tumors on mammography, DCE, MRI, and ultrasound tests from radiomic features. Radiomics has been applied to many diseases, including cancer and neurodegenerative diseases, to name a few [32]. Convolutional neural networks have also been applied to segment organs at risk. Furthermore, they can predict the response to neoadjuvant chemoradiation therapy assessed at the time of surgery for non-small cell lung cancer (NSCLC) and locally advanced rectal cancer.

The radiomic method in combination with Machine Learning and Deep Learning requires several steps, the first of which is the pre-processing step that must be applied to the image to reduce image noise and improve image quality, enabling reproducible and comparable radiomic analyses. Image smoothing can be achieved with average or Gaussian filters. The resampling of voxel sizes is important for data sets with variable voxel sizes [18,32,33,34].

There are two types of interpolation algorithms, polynomial interpolation, and spline interpolation. The polynomial method is a zero-order polynomial method that assigns the gray level values of the nearest neighbors to the interpolated point. For two- and three-dimensional interpolation, the following methods are used: bilinear or trilinear interpolation and bicubic or tricubic interpolation. Polynomial methods of the third type are cubic spline interpolation and convolutional interpolation, which interpolate more uniform surfaces than the linear method. Linear interpolation is a commonly used algorithm, as it does not produce the coarse blocking artifacts generated by the nearest neighbors, nor the out-of-range gray levels that might be produced by higher-order interpolation.

In the context of feature-based radiometric analysis, the calculation of textures requires the discretization of gray levels, which can be done in two ways: fixed number of bins N and fixed width of bins B.

When the mode used is not well calibrated, it is better to fix the number of bins; this technique maintains contrast but loses the relationship between image intensities.

Formally, by fixing a number of N bins, the gray levels will be discretized in these bins using the following formula:

(4) $$\left\{\begin{array}{cc} \left[N_{g}\frac{X_{gl,k}-X_{gl,min}}{X_{gl,max}-X_{gl,min}}\right]+1 & X_{gl,k}<X_{gl,max}\\ N_{g} & X_{gl,k}>X_{gl,max} \end{array}\right.$$

where $X_{gl,k}$ is the intensity of the k-th voxel.

The fixed bins method, on the other hand, maintains a direct relationship with the original scale. It can be formalized from a minimum $X_{gl,k,min}$, and a new bin will be assigned for each intensity interval of $w_{b}$.

The gray levels are discretized as follows:

(5) $$X_{d,k}=\left[\frac{X_{gl,k}-X_{gl,min}}{w_{b}}\right]+1$$

Investigations into the effect of both methods have shown differing effectiveness, with the fixed bin size method offering better repeatability; this is currently the subject of research.

Methods based on radiomic features require segmentation of the ROI by a manual procedure performed by the radiologist or an automatic or semi-automatic procedure performed by software to segment the lesion. Extracted radiomic features are hand-created features that capture characteristic patterns in the imaging data, including shape-based features such as the shape, size, and surface information of the ROIs.

There are different types of features that describe the qualitative image features typically used in the radiological workflow. Once the subset of features has been identified, various Machine Learning algorithms can be applied.

Selection is, therefore, a fundamental step that aims to optimally represent the features that correlate most with the endpoint and, at the same time, correlate least with each other.

First-order features describe the intensities and overall variations in ROIs. Second-order (texture) features can provide interrelationships between voxels. Textural features can be extracted from different matrices, such as the gray-level co-occurrence matrix (GLCM), the gray-level run-length matrix (GLRLM), etc. [32,34].

Sometimes, feature selection and model construction can be implemented together; these are called embedded methods. Differently, wrapper methods select features based on the performance of models for different subsets of features so that the model has to be rebuilt again after the features have been selected. The filter method also separates the processes of feature selection and model construction, the uniqueness of which is the independence of the classifier used for subsequent model construction. These three feature selections are mostly supervised, while unsupervised methods include principal component analysis (PCA), clustering, and t-distributed stochastic neighbor embedding (t-SNE).

Although the handcrafted features introduced above provide preliminary knowledge, they may not faithfully capture the underlying imaging information. To solve this problem, the following can be used: Deep Learning technologies based on multilayer neural networks, in particular, convolutional neural networks (CNNs), whose architectures have been modified to suit the specifications of large-scale imaging. Inspired by Hubel and Wiesel’s work on the animal visual cortex.

## 3. Radiomics

The application of AI in medicine is a fascinating journey in constant evolution. For years, through simple visual analysis, we have been able to identify the presence of a tumor and to qualitatively define its precise anatomical location, morphological characteristics, margins, and its extent in the surrounding structures, but, for example, we cannot predict whether the tumor will respond to cancer therapy. Visual analysis is estimated to be able to extract only about 10% of the information contained in a digital mammografic image [35].

In addition to the classic images, the new diagnostic technologies produce an enormous wealth of numerical data that simple visual observation, or qualitative analysis, cannot process. A qualitative analysis supported by radiomics is, therefore, essential. Specifically, radiomics in mammography is a field of study that aims to extract a large number of quantitative features from medical images, using data characterization algorithms. In fact, the mammography images are composed of pixels, analyzed in detail by powerful computers through complex mathematical algorithms, and converted into numerical data, which are then processed, in very large numbers, by dedicated calculation tools. The manipulation and analyses of large amounts of data require the use of advanced techniques for their management. These techniques make it possible to synthesize data, obtaining quantitative information objective for clinical decision support, capable of providing details on the underlying physio-pathological phenomena inaccessible to simple visual analysis. The results thus obtained guarantee a better clinical approach to the patient: the integration of anamnestic data with pathological and histological data allows the production of predictive models on the patient’s prognosis, on the response to therapy, and on the appropriate follow-up [36].

Radiomic can be applied to most imaging modalities in the breast study such as tomosynthesis, MRI, or ultrasound, and it is based on the creation of a database of quantitative characteristics, which requires following steps [37,38]:

- Image acquisition in standardized mode;
- Segmentation of areas or volumes of interest;
- Extraction and categorization of descriptive characteristics;
- Integration of extracted quantitative data with quantitative data obtained from other sources;
- Construction of predictive models.

First, after having acquired the images in standardized mode, they are processed through the use of a variety of reconstruction algorithms, such as contrast or edge enhancement. The images are processed in order to increase their quality and readability to improve the ease and precision with which an anomalous feature can be detected and characterized. Subsequently, the segmentation of the image takes place. On mammographic images, once a characteristic finding has been recognized such as a mass or a group of calcifications, areas or volumes of interest are identified [34].

**Figure 2:**
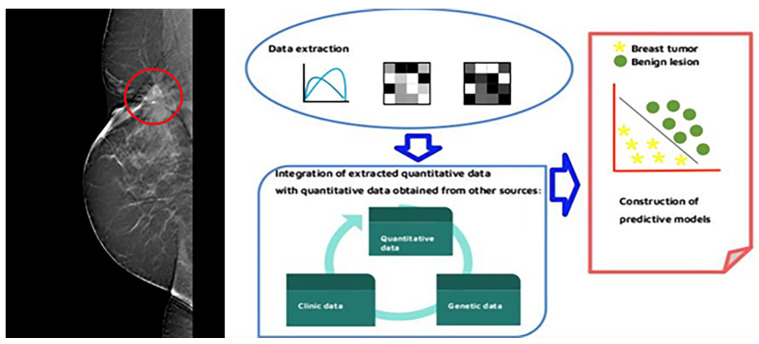
The radiomics analysis simplification.

This is usually done through manual processes, or, alternatively, it is possible to rely on semi-automated or fully automated processes, using artificial intelligence, especially for large data sets. The characteristics of the find are extracted from the aforementioned area of interest, which include volume, shape, surface, density, intensity, position, and relationships with the surrounding tissues, Ref. [36] but not only as represented in Figure 2.

In radiomics, in fact, two types of characteristics are distinguished:

1. Semantic characteristics: commonly used in the radiological lexicon to describe regions of interest.
2. Agnostic characteristics: they attempt to capture the heterogeneity of the lesion through quantitative mathematical descriptors.

From a specific area of interest, we can extract the characteristics of the find, which we can distinguish between semantic and agnostic, as we can see in the Table 1.

The semantic characteristics are those that are used in drafting a radiological report and are widely standardized to describe the regions of interest such as, for example, the shape, the margins, the density of a mass, the morphology and distribution of the calcifications, etc.

The agnostic characteristics, on the other hand, are used to define and analyze the heterogeneity of the lesion by applying quantitative mathematical descriptors, such as, for example, the use of histograms for the evaluation of asymmetry and kurtosis, Laplacian transformations, and, above all, the texture analysis.

Therefore, a region of a mammographic image can be represented by its external characteristics or by its internal characteristics, such as through texture analysis. In radiology and, precisely, in the study of the breast cancer patient, it is assumed that intratumoral heterogeneity is a marker of malignancy; texture analysis attempts to provide a comprehensive quantitative analysis of heterogeneity through the evaluation of pixels and voxels within a tumor image [39,40].

Texture analysis is a mathematical method. The use of texture analysis represents a non-invasive method to identify and characterize lesions using imaging, improving the results in terms of characterization [41].

Through the management of digital medical images in the form of large data matrices, it aims to extract a large number of morphological and predictive characteristics, aimed at evaluating their spatial heterogeneity in the regions of interest. It generally shows how the numerical values present within the images are spatially distributed within the studied tissue, Refs. [39,42], indispensable in the evaluation of a region of interest. Texture characteristics are obtained from segmented ROIs on the images. Even if it is not a new research topic, the last decade has seen a significant resurgence of texture analysis in the field of radiomics [39,41].

Specifically, texture analysis analyzes the relationships, the so-called “descriptors”, between the pixels or voxels that make up the image. It employs a plethora of models to obtain an accurate assessment of the heterogeneity of radiological findings. The use of descriptor-based models is the most common in texture analysis and involves three orders: first-order descriptors, second-order descriptors, and higher-order descriptors. The first order descriptors are computed from the native values of the image. The calculation is performed starting from the histogram, which, on the abscissa, shows the single gray values present in the studied region, while, on the ordinate, the number of times these appear in it. They demonstrate the frequency of the distribution in the region of interest by means of a histogram, not considering the pixels around the region of interest. First-order statistics measure parameters such as intensity, standard deviation, asymmetry, and kurtosis [39,42].

Descriptors of second, third, or higher orders are defined as descriptors that analyze the relationships between two or more pixels/voxels of the image, according to the number of elements taken into consideration. The images are not analyzed directly, but matrices are obtained that are then analyzed using appropriate descriptors. There are different matrices, for example, the Gray Level Co-occurrence Matrix (Matrix Co-occurrence often abbreviated as GLCM) can give the definition of Haralick features that reflect the frequency of pixels with equal intensities of gray levels appearing consecutively at a certain distance (offset) or the frequency that pairs of predetermined pixel values that occur within a spatial interval in the image. In this way, co-occurrence measurements are obtained, which provide information regarding the plot of the region of interest. Higher-order statistics provide a broader overall ratio of the region-of-interest plot metrics [39,42]. They explore the overall differences between pixels or voxels in the context of the entire region of interest, often through the use of the gray tone difference matrix. Another classic technique that extracts features of higher order statistics is a statistic called matrix RLM (Gray-Level Run Length Matrix, Matrix of the length of the run of the gray levels, abbreviated as GLRLM) where “run” means the presence of elements in image with the same intensity of gray in a row. This matrix will then report how often in the image it is possible to observe “rows” of elements with a certain length and with a certain intensity. From the complex procedures described above, a set of features is obtained, the different characteristics of the region of interest. Subsequently, the classification takes place, i.e., the operation of assigning an individual to a specific class in relation to the extracted features. The individual may be a single lesion or a patient. Precisely, considering an individual and constructing its vector of features, a classifier is an algorithm for assigning this individual to a class among n possible classes. The classification techniques can be divided into linear techniques that adopt a linear combination (sum) of the features to try to classify the individual and into non-linear techniques such as trees (trees), which are based on successive dichotomous processes: at each step, the algorithm creates a binary separation, further split in two in the next step. The quantitative data thus obtained are subsequently integrated with other quantitative data from other sources, such as those obtained from laboratory and clinical data and from genomic tests. Thus, it is possible to study the correlation between the data obtained from the images and the molecular characteristics of the tumor or to obtain new information on the tumor phenotype and the microenvironment (see Figure 2) [43,44].

Therefore, prediction models are generated: the final aim is extracting directly from the images the indications on the aggressiveness of the disease, the most suitable therapies, and the possible response to treatment [45]. Currently, through biopsy analysis, we are able to obtain information on a single fragment of a lesion, a fragment of tissue. On the other hand, with the data obtained through the use of radiomics, we reach the advantage of collecting characteristics of an entire area or lesion. If we think of the digital mammographic image as constituted by millions of voxels and pixels, the lesion area, consequently, contains hundreds of quantitative characteristics objective for clinical decision support, capable of providing details on the underlying physio-pathological phenomena, inaccessible to simple visual analysis [43,44,45].

Furthermore, the synthesis of radiomic data with genetic data has allowed the development of a further field of study: radiogenomics. The combination of the aforementioned data allows us to obtain information on the characteristics and subtypes of a tumor and to direct the most appropriate therapeutic choice [20,46,47].

**Figure 3:**
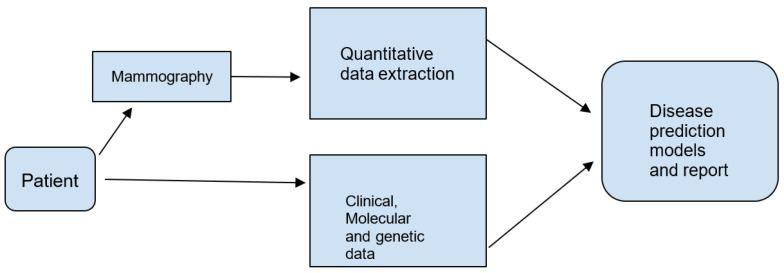
The radiomics flowchart.

**Figure 4:**
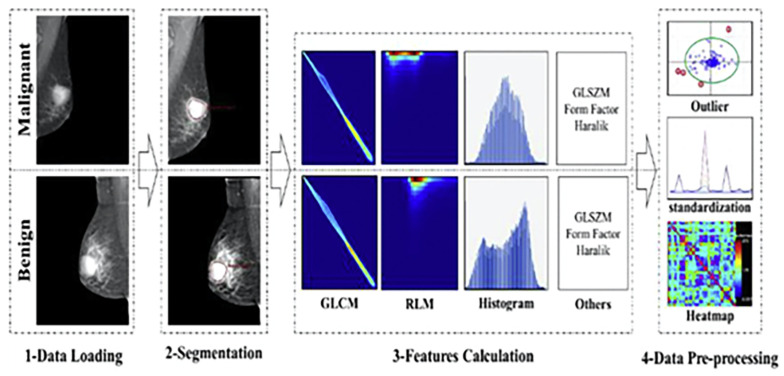
The radiomics workflow of acquisition and pre-processing [46].

Diagrams summarising the radiomics flowchart Figure 3 and the radiomics acquisition and pre-processing flowchart Figure 4 can be seen in the following figures.

Recent studies suggested that the application of radiomics in mammography could produce significant results in the diagnosis of breast cancer. In 2019, in the study by Li et al. [48], the extraction of quantitative features from the analysis of the mammograms of 182 patients proved to be better than simple qualitative analysis of the lesion [49]. Furthermore, from the study by Tagliafico et al. [50], also conducted in 2019, on 40 patients with high breast density, it was shown that the use of radiomics in tomosynthesis was useful in differentiating malignant findings from normal breast tissue [49]. Mao et al. [51], in a 2021 multicenter study, demonstrated with a AUC of 0.92 how the application of the radiomics model in mammography could predict the risk of breast cancer [49]. Woodard et al. [52], in 2018, showed how BI-RADS descriptors could assume the role of imaging biomarkers for breast cancer through the analysis of radiomic qualitative features obtained from the correlation between the genomic tests used in clinical practice, OncotypeDX recurrence risk scores, and the semantic characteristics used in the lexicon of mammography [53].

However, the rapid advancement of AI in medical imaging has identified a number of challenges to address in building a robust and reliable process [23,35,47]. Among the most critical, the problem of responsibility certainly falls [30,54]. In this regard, the medico-legal question that then arises is the question of “who is responsible for the diagnosis”, especially if it is wrong [20,31,55].

Whether the data scientists or manufacturers involved in the development, marketing, and installation of artificial intelligence systems will have the ultimate legal responsibility for the negative results resulting from the use of the artificial intelligence algorithm is a difficult legal question; if physicians are no longer the primary agents of the interpretation of radiological studies, will they still be held accountable? If radiologists monitor the results of the AI system and still have a role in validating the AI interpretations, do they still have ultimate responsibility, even if they do not understand and cannot question the precise means by which a diagnosis was determined? This poses many challenges, not least the basic human need to understand how and why important decisions have been made [31,55].

Mistakes occur when the radiologist accepts or mistakenly implements a machine’s decision despite evidence to the contrary. The typical characteristic of the action performed by the radiologist, as a professional, is the autonomy in the decisions regarding the provision of the service, the technical tools to be used, and the personality of the service [18].

In a recent article published by Thomas PS et al., the application of a Seldon algorithm was described. The authors proposed a framework to design Machine Learning algorithms and show how it could be used to build algorithms that provided its users with the ability to easily place limits on the likelihood that the algorithm would produce any specified unwanted behavior. Perhaps the solution is to create an ethical AI, subject to constant control of action, as it indeed happens for human conscience: an AI subject to indirect civil liability, written in the software and that manufacturers must guarantee to users. Thus, they can use AI sensibly and with human-controlled automation. It is clear that future legislation must outline the contours of the professional’s responsibility, with respect to the provision of the service performed independently by AI, balancing the professional’s ability to influence and, therefore, correct the machine, limiting the sphere of autonomy that technological evolution would recognize as robotic [30].

## 4. Functional Imaging

Nuclear Medicine is ideally placed for the development of Radiomics applications [56,57]. In fact, Nuclear Medicine images have the ability to correlate morphological data with functional data [58,59]. By marking the main molecules present in the body, it is possible to trace the biological functions such as metabolism [60], blood flow [61], membrane permeability [62], and receptor status [63]. The images acquired with the SPECT or PET technique are then processed with dedicated software to extract numerical parameters, curves, and parametric images [64].

Despite the limitations due to low spatial resolution, in the field of nuclear medicine, radiomics is developing as an equal to traditional radiology, taking advantage of the information functional that this can offer. In particular, in the radiomic scope, PET is the most promising test, and several studies suggested that PET acquisition radiomics analysis could provide a valid support in the diagnosis, staging, and characterization of various tumors [65].

Nuclear imaging for disease evaluation tie such as cancer and other non-malignant diseases is increasingly used to explore the disease phenotype and to support re-individualized clinical decision-making on the road to personalized medicine [66]. One of the biggest challenges promising for radiomics analysis on PET imaging in breast cancer is the prediction of Pathological Complete Response (pCR) to chemotherapy treatment neoadjuvants, as this is an important factor prognostic. Several studies have also demonstrated a significant correlation between Image-Based Bio-Markers and histology, as well as significant associations with receptors.

## 5. Conclusions

The breast is an extremely complex organ that varies from patient to patient, characterized by a structure that is difficult to explore only with diagnostic imaging techniques. The difficulty in exploring the whole corpus mammae determines an increase in the incidence of errors in the detection of breast cancer, especially when the characteristics of it overlap with benign formations, mimicking their morphological aspects. The instrumental identification of neoplasia is increasingly based on minimal and nuanced signs, for which just the histological definition is conclusive, to date. With these assumptions, it is clear that breast diagnostics need not only to be entrusted to experienced professionals assisted by equipment with high quality standards but also, and above all, to integrate with radiomics as a tool for analyzing the breast structure. Thus, the commitment in the study of radiomics, in the automated analysis of the structure of mammary parenchyma, gets stronger, with the aim of discovering the characteristics of the disease that could not be identified through a simple human visual observation. In fact, through imaging, we will be able to identify phenotypes at risk for breast cancer, suggest more appropriate screening routines, accelerate individualized risk stratification in breast cancer prevention strategies, aid in the detection of cancers, identify women who may benefit from additional examination, reveal specific biomarkers through the correlation between the characteristics of the tumor extracted from medical images and gene expression in the radiogenomics branch, and, furthermore, by means of image texture analysis software, aim to differentiate a priori and with good accuracy the patients who will respond to the therapy from those in which the disease will progress. Radiomics arouses great interest in the breast medicine world due to its numerous improvements that it proposes to get in the fight against breast cancer not only for the patient’s health but also for the doctor’s work. Future work is full of technical challenges in this field, which is still under study and is exposed to various limitations in its application, but large prospective studies should aim to improve the fight against breast cancer in clinical practice.

## Figures and Tables

**Table 1:** Semantic and agnostic characteristics.

Semantic Features	Agnostic Features
Location	Histogram (asymmetry, kurtosis)
Form	Laplacian transformations
Margins	Haralick Texture, Law Texture
Vascularization	Fractal dimensions
